# Model Predictive Control for Speed-Dependent Active Suspension System with Road Preview Information

**DOI:** 10.3390/s24072255

**Published:** 2024-04-01

**Authors:** Qiangqiang Li, Zhiyong Chen, Haisheng Song, Yahui Dong

**Affiliations:** 1 The State Key Laboratory of Automotive Simulation and Control, Jilin University, Changchun 130012, China; qqli20@mails.jlu.edu.cn; 2 The Vehicle and Traffic Engineering, Henan Institute of Technology School, Zhengzhou 453003, China; songhs09@sina.com; 3 Shaanxi Heavy Duty Automobile, Xi’an 710299, China; dyhjy2017@163.com

**Keywords:** active suspension, linear parameter varying (LPV), model predictive control (MPC), road preview information

## Abstract

This paper proposes a model predictive control (MPC) scheme based on linear parameter variation to enhance the damping control of speed-dependent active suspensions. The controller is developed by introducing a speed-dependent term, specifically front- and rear-wheel time delays, to the half-car model using the Padé approximation. Subsequently, the model is augmented with time-varying parameter dependence. An adaptive Kalman filter based on variance matching is employed to estimate system states affected by imprecise sensor measurement noise. Finally, a set of explicit control laws incorporating road preview information and available vehicle speed are determined offline using multi-parameter linear programming (mp-LP), simplifying online implementation to searching for optimal solutions in a lookup table. Simulation results demonstrate a significant improvement in active suspension control under changing vehicle speeds compared to passive control.

## 1. Introduction

The suspension system is pivotal for vehicle performance, providing both support and vibration reduction. Automotive research has focused extensively on suspension control, which is broadly categorized as passive, semi-active, and active [1,2,3]. Active suspensions, characterized by controllable performance and rapid adjustment, have garnered attention for their potential to enhance vehicle comfort. However, the control force of active suspensions is constrained, underscoring the significance of the control strategy in optimizing system performance. This paper addresses the control of an active suspension system dependent on vehicle speed to minimize the vehicle vibration response.

The integration of forward-looking sensors, such as radar and cameras, in automotive research has facilitated accurate road information collection [4]. Predictive control technology, leveraging available road information, enhances suspension damping performance. Theoretical frameworks, including Wiener filter theory [5] and dynamic programming methods [6], support the design of suspension controllers integrating predictive information. While the linear–quadratic regulator (LQR) and model predictive control (MPC) are common approaches, the LQR often overlooks system constraints [7,8], while MPC offers promise for suspension control with predictive road information [9,10].

The MPC approach requires the system state to be fully measurable, but the measurements are subject to sensor uncertainty and unknown road conditions, making it impossible to measure the system state variables accurately. For example, in [11], an MPC scheme that uses on-board sensors was designed for quarter-car models to pre-collect the road profile ahead and use it to predict the controller output. However, the controller design process ignores the confounding effect of sensor measurement noise in the prediction domain on the system state observation. Therefore, in [12,13,14], a number of state-measurement-based MPC methods are proposed to improve the ride comfort of the vehicle. Kalman filtering is a recursive state-space model based on optimal estimation and is widely used for the state estimation of dynamic systems, such as positioning, tracking, and control [15]. In the state estimation process, the performance of the filter mainly depends on the accuracy of the noise covariance, and an inaccurate noise covariance can lead to estimation errors and even filter divergence [16]. To estimate states with time-varying noise covariances, this paper constructs an estimator using covariance matching techniques. The covariance matching method uses a sliding window of residual series to approximate the true noise covariance. Due to the simplicity of the implementation process, it has been widely used in many studies [17,18,19].

Furthermore, most studies only consider linear time-invariant (LTI) vehicle models, which are not fully representative of the dynamic characteristics of the vehicle system. The model-based linear parameter-varying (LPV) approach is often more efficient when there is non-stationary behavior in the system model. The formulation of LPV systems is usually based on a bounded and known control variable [20]. Note that the parameter can only be measured or estimated at the current time, while the behavior at future times is usually unknown. However, most studies have only considered robust control strategies. In [21], the influence of the suspension system on the parameters of the spring-loaded mass with uncertainty is overcome by $H_{\infty }$-robust control, but the obvious disadvantage of this type of robust approach is that it is overly conservative, as the controller output must always consider the worst-case optimal solution. In [22], the variation in the model parameters due to changes in vehicle speed during actual vehicle driving is considered. To fully account for the effect of time-varying vehicle speed on the suspension controller during vehicle travel, a linear time-varying semi-vehicle-based model was designed, and sensor measurement noise disturbances and road-ahead information were considered in the controller design process. In summary, this paper makes the following contributions:

- The utilization of the Padé approximation to describe the delay characteristics of front- and rear-wheel road inputs, rewriting the half-car model as an augmented model with respect to time-varying parameters.
- The adoption of the variance matching method to construct an adaptive Kalman filter for the online estimation of the system state under the influence of sensor measurement noise and the adjustment of the variance matrix of measurement noise.
- The proposal of an MPC-LPV control method with available road preview and vehicle speed information inputs. Within the LPV model, the iterative optimization of the cost function of the prediction domain using an mp-LP technique enables the offline computation of an explicit MPC-LPV control law and online lookup table search.

The remainder of the paper is structured as follows: Section 2 outlines the augmented half-car model according to the Padé approximation. Section 3 introduces the proposed MPC-LPV controller. Subsequently, Section 4 presents different simulation scenarios designed to verify controller performance. Finally, conclusions are drawn in Section 5.

## 2. Problem Formulation

### 2.1. Suspension Model

**Figure 1:**
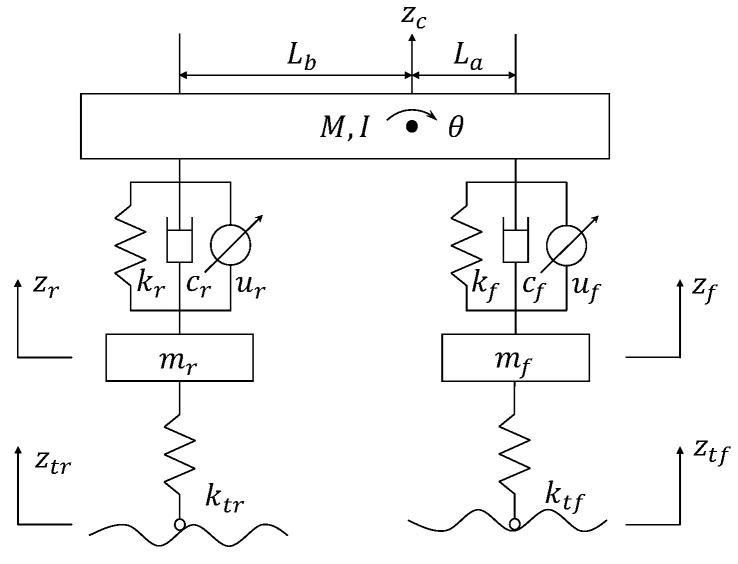
Half-car suspension system.

In previous studies, several sophisticated suspension models have been proposed to simulate the intricate dynamic characteristics of suspension systems [23]. In this study, we employ a half-car (4-DOF) model to analyze the suspension dynamic response [24]. This model offers an effective representation of both the vertical and pitch motions of a vehicle, thereby closely mirroring the actual operational state of the vehicle in comparison to the quarter-car model. Such fidelity to real-world vehicle dynamics enhances its utility in assessing the efficacy of control systems aimed at enhancing vehicle stability and maneuverability. The structure of the suspension model is illustrated in Figure 1.

According to Newton’s second law, the dynamic equations of the suspension system are given by

(1) $$\begin{array}{c} M{\ddot{z}}_{c}=F_{f}+F_{r}\\ I\ddot{\theta }=L_{a}F_{f}-L_{b}F_{r}\\ m_{f}{\ddot{z}}_{f}=-k_{tf}\left(z_{f}-z_{tf}\right)-F_{f}\\ m_{r}{\ddot{z}}_{r}=-k_{tr}\left(z_{r}-z_{tr}\right)-F_{r} \end{array}$$

where $F_{f}$ and $F_{r}$ represent the front and rear suspension forces at the sprung mass, respectively. They can be expressed as follows:

(2) $$\begin{array}{c} F_{f}=k_{f}\left(z_{f}-z_{c}-L_{a}\theta \right)+c_{f}\left({\dot{z}}_{f}-{\dot{z}}_{c}-L_{a}\dot{\theta }\right)+u_{f}\\ F_{r}=k_{r}\left(z_{r}-z_{c}+L_{b}\theta \right)+c_{r}\left({\dot{z}}_{r}-{\dot{z}}_{c}+L_{b}\dot{\theta }\right)+u_{r} \end{array}$$

In the dynamic system above, M,I, and θ are the sprung mass, the rotational inertia, and the pitch angle of the car body, respectively. $L_{a}$ and $L_{b}$ are the wheelbases of the front and rear wheels, respectively. $z_{c},z_{c}+L_{a}\theta$, and $z_{c}-L_{b}\theta$ are the absolute displacements of the sprung mass, front axle, and rear axle, respectively. $z_{f}$ and $z_{r}$ are the absolute displacements of the front unsprung mass and rear unsprung mass. $m_{f}$ and $m_{r}$ are the front and rear unsprung masses, $z_{tf}$ and $z_{tr}$ are the front and rear road inputs. $k_{f},k_{r}$, and $c_{f},c_{r}$ are the passive suspension stiffness and damping coefficients, and $k_{tf}$ and $k_{tr}$ are the front and rear tire stiffness. $u_{f}$ and $u_{r}$ represent the active control forces of the front and rear suspensions, respectively.

The state variable vector for the half-car vehicle model is chosen as

(3) $$\begin{array}{c} x={\left[x_{1},x_{2},x_{3},x_{4},x_{5},x_{6},x_{7},x_{8}\right]}^{T} \end{array}$$

where

$$\begin{array}{c} x_{1}=z_{c}+L_{a}\theta ,x_{2}=z_{c}-L_{b}\theta ,x_{3}=z_{f},x_{4}=z_{r},\\ x_{5}={\dot{z}}_{c}+L_{a}\dot{\theta },x_{6}={\dot{z}}_{c}-L_{b}\dot{\theta },x_{7}={\dot{z}}_{f},x_{8}={\dot{z}}_{r} \end{array}$$

In addition, the controlled input forces of the active suspension and the road inputs to the suspension system are described as follows:

(4) $$\begin{array}{c} u=\left[\begin{array}{c} u_{f}\\ u_{r} \end{array}\right],d=\left[\begin{array}{c} z_{tf}\\ z_{tr} \end{array}\right] \end{array}$$

In this paper, three main ride comfort evaluation indicators are selected as system outputs of the half-car model: (1) sprung mass acceleration $y_{1}={\ddot{z}}_{c}$ and pitch acceleration $y_{2}=\ddot{\theta }$; (2) front and rear dynamic spring deflection $y_{3}=z_{c}+L_{a}\theta -z_{f},y_{4}=z_{c}-L_{b}\theta -z_{r}$; (3) and dynamic front/rear wheel to road loads $y_{5}=k_{tf}\left(z_{f}-z_{tf}\right),y_{6}=k_{tr}\left(z_{r}-z_{tr}\right)$.

The equation of the continuous-time state-space model can be written as

(5) $$\begin{array}{cc} {\dot{x}}_{c}\left(t\right) & =A_{c}\;x_{c}\left(t\right)+B_{c,u}\;u\left(t\right)+B_{c,d}\;d\left(t\right)\\ y(t) & =C_{c}\;x_{c}\left(t\right)+D_{c,u}\;u\left(t\right)+D_{c,d}\;d\left(t\right) \end{array}$$

where $A_{c}$, $B_{c,u}$, $B_{c,d}$, $C_{c}$, $D_{c,u}$, and $D_{c,d}$ are the parameter matrices of the state-space equations, which are specifically defined in Appendix A.1.

There are three areas of performance constraints that need to be considered when designing controllers for active suspension systems:

1. Suspension deflection control: As the suspension system is located between the vehicle body and the wheels, there are structural constraints on the mechanical components. Therefore, the suspension deflection limit must be considered in the controller design process to avoid bottom-out damage caused by excessive operation, i.e.,
  (6) $$\begin{array}{c} |z_{c}+L_{a}\theta -z_{f}|\leq SS_{max}\\ |z_{c}-L_{b}\theta -z_{r}|\leq SS_{max} \end{array}$$
  where $SS_{max}$ is the limiting constraint amount that the suspension movement must not exceed.
2. Road-holding limit: In order to ensure driving safety, it is necessary for the wheel to maintain permanent firm contact with the road, which is also described in some of the literature [25,26]: i.e., the dynamic load of the tire should not exceed the static load.
  (7) $$\begin{array}{c} k_{tf}\left(z_{f}-z_{tf}\right)\leq \left(\frac{L_{b}M}{L_{a}+L_{b}}+m_{f}\right)\;g\\ k_{tr}\left(z_{r}-z_{tr}\right)\leq \left(\frac{L_{a}M}{L_{a}+L_{b}}+m_{r}\right)\;g \end{array}$$
  where *g* is the acceleration of gravity.
3. Actuator Limit: Due to the limited drive force provided by the actuator, there is an obvious saturation limit, so the active control force should not exceed a certain limit, i.e.,
  (8) $$\begin{array}{c} \left|u\right|\leq u_{max} \end{array}$$

where $u_{max}$ is the maximum control force provided by the active suspension actuator.

### 2.2. Augmented System Formulation

In the half-car model, since the lateral motion of the vehicle is not considered, it can be assumed that when the rear wheels of the vehicle roll along the road trajectory driven by the front wheels, there is only a time delay τ in the wheelbase length between the road inputs to the front and rear wheels. The relationship between the front-wheel road excitation and the rear-wheel excitation is therefore as follows:

(9) $$\begin{array}{c} z_{r}\left(t\right)=z_{f}\left(t-\tau \right) \end{array}$$

(10) $$\begin{array}{c} \tau =(L_{a}+L_{b})/v \end{array}$$

where *v* represents the vehicle speed.

The relationship between $z_{f}\left(t\right)$ and $z_{r}\left(t\right)$ is expressed in terms of the Laplace transfer function:

(11) $$\begin{array}{c} L\left[\frac{z_{r}\left(t\right)}{z_{f}\left(t\right)}\right]=e^{-\tau s} \end{array}$$

By choosing the Padé approximation, $e^{-\tau s}$ can be rewritten as a linear polynomial function of order *m* [27], i.e.,

(12) $$\begin{array}{cc} e^{-\tau s} & =\frac{\sum_{j=0}^{m}\frac{(2m-j)!m!}{(2m)!j!(m-j)!}{\left(-\tau s\right)}^{j}}{\sum_{j=0}^{m}\frac{(2m-j)!m!}{(2m)!j!(m-j)!}{\left(\tau s\right)}^{j}}\\ \; & =\frac{\beta_{0}+\beta_{1}\left(-\tau \right)s+\cdots +\beta_{m}{\left(-\tau \right)}^{m}s^{m}}{\beta_{0}+\beta_{1}\tau s+\cdots +\beta_{m}\tau^{m}s^{m}} \end{array}$$

where

$$\begin{array}{c} \beta_{j}=\frac{(2m-j)!}{j!(m-j)!},j=0,1,\ldots ,m. \end{array}$$

Then, the state vector is chosen as $x_{\xi }=\left[\xi ,\tau \dot{\xi },\ldots ,\tau^{\left(m-1\right)}\xi^{\left(m-1\right)}\right]$, and the inverse Laplace transform of (12) can be obtained:

(13) $$\begin{array}{cc} {\dot{x}}_{\xi }\left(t\right) & =A_{\xi }\;x_{\xi }\left(t\right)+B_{\xi }\;z_{f}\left(t\right)\\ z_{r}\left(t\right) & =C_{\xi }\;x_{\xi }\left(t\right)+D_{\xi }\;z_{f}\left(t\right) \end{array}$$

where the parameter matrix for the state space (13) is given in Appendix A.2 ($A_{\xi }$, $B_{\xi }$, $C_{\xi }$, and $D_{\xi }$).

**Figure 2:**
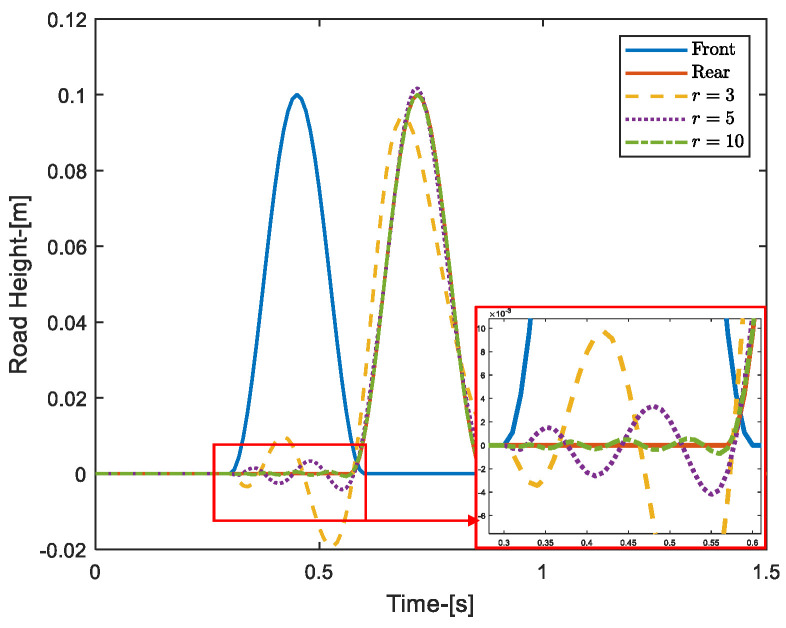
Example of Padé approximation.

Here, we take a collision excitation signal as an example, assuming that the actual input to the front wheel is known, as shown in Figure 2. By comparing the approximate effect of the rear-wheel input with different values of *m*, it can be seen that the larger the *m*, the closer to the real situation.

In [28,29], the wheel input can usually be assumed to be a linearly filtered model consisting of Gaussian white noise, so the front-wheel input to the vehicle has the following form:

(14) $$\begin{array}{cc} {\dot{z}}_{f}\left(t\right) & =-2\pi n_{1}vz_{f}\left(t\right)+2\pi n_{0}\sqrt{G_{q}\left(n_{0}\right)v}w\left(t\right) \end{array}$$

where $n_{0}$ is the reference spatial frequency, typically $n_{0}$ = 0.1 $m^{-1}$. $n_{1}$ is the lower cut-off spatial frequency of the road excitation, typically $n_{1}$ = 0.01 $m^{-1}$. $G_{q}\left(n_{0}\right)$ is the power spectral density of the road at the reference spatial frequency, and w(t) is the unit variance Gaussian white noise process.

Finally, we choose $x\left(t\right)={\left[x_{c}\left(t\right),x_{\xi }\left(t\right),z_{f}\left(t\right)\right]}^{T}$ as the state vector for the augmented system and then obtain the augmented system form of the half-car model.

(15) $$\begin{array}{cc} \dot{x}\left(t\right) & =A\left(\tau \right)\;x\left(t\right)+B_{u}\;u\left(t\right)+B_{d}\left(\tau \right)\;w\left(t\right)\\ y(t) & =C\;x\left(t\right)+D_{u}\;u\left(t\right)+D_{d}\;w\left(t\right) \end{array}$$

where the augmented system parameter matrix is given in Appendix A.3 (*A*, $B_{u}$, $B_{d}$, *C*, $D_{u}$, and $D_{d}$). Note that the affine relation with the system matrix (*A*, $B_{d}$) is not formed by τ, but by the parameter $\frac{1}{\tau }$. In contrast to the half-car model in (5), which relies on road inputs from the front and rear wheels $z_{tf},z_{tr}$, in the extended system, the road input terms *d* are converted to Gaussian white noise *w*, where the parameter matrices *A* and $B_{d}$ depend on the speed-dependent term τ.

## 3. Main Methods

**Figure 3:**
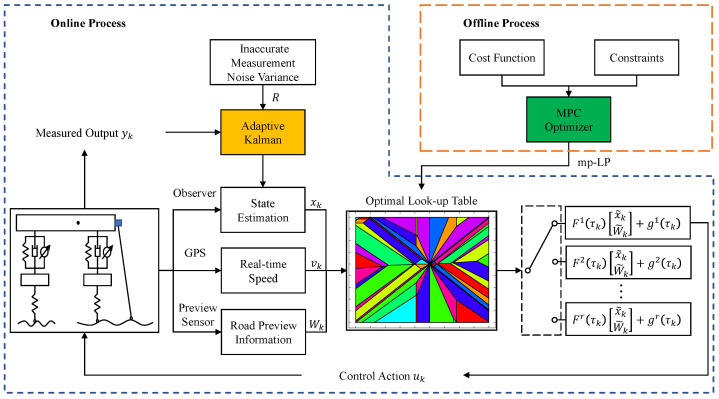
The flow chart of the proposed MPC-LPV model.

In this section, we consider the changes in the parameters of the model matrix due to changes in vehicle speed based on the augmented model after the Padé approximation. We will address this issue within the LPV framework. For this purpose, Figure 3 shows the general architecture of the MPC-LPV proposed in this paper. The proposed MPC-LPV consists of two phases, an offline phase and an online phase. The output of the offline phase is a lookup table for the optimal control output; i.e., a cost function containing the future prediction domain $N_{p}$ and the control domain $N_{c}$ is designed in the feasible domain of the scheduling parameters, and the optimization problem is solved offline using the mp-LP method. In the online phase, an adaptive Kalman estimator based on a sequence of residuals is first designed to estimate the full state of the system by adjusting the imprecise measurement noise variance, and finally, the optimal control output is calculated by searching the lookup table based on the available input information, such as the current system state, the road ahead, and the vehicle speed.

### 3.1. System Prediction

Due to the reliable development of vehicle sensor technology at this stage, road profile information can be accurately obtained. Therefore, the design of the suspension controller assumes that the vertical road profile is acquired by a LiDAR system [30] or camera [31] mounted on the front of the vehicle and stored in a displacement register with a memory size of N×1. For this, the content *W* of the stored road preview information can be read by the front-wheel input as

(16) $$W\left(t\right)=\left[\begin{array}{c} z_{f}\left(t\right)\\ z_{f}\left(t+1\right)\\ \vdots \\ z_{f}\left(t+N-1\right) \end{array}\right]$$

Furthermore, it is important to note that, while our assumed conditions for the execution of the MPC-LPV controller include the availability of sufficient preview information, it is crucial to recognize that inadequate road surface preview information within the register can potentially degrade the performance of MPC strategies that consider preview information [32]. Therefore, careful consideration must be given to the adequacy and accuracy of preview information to ensure optimal closed-loop system performance.

In this paper, it is assumed that vehicle speed measurements are available in real time and vary within the interval $v\in [v_{\min},v_{\max}]$. In (10), the time delay τ is linearly related to the vehicle speed *v*, so τ also varies with the vehicle speed during the actual driving of the vehicle. The time delay τ is therefore subject to the following constraint:

(17) $$\begin{array}{c} \tau_{1}\leq \tau \leq \tau_{2} \end{array}$$

where $\tau_{1}=\frac{L_{a}+L_{b}}{v_{\max}}$ and $\tau_{2}=\frac{L_{a}+L_{b}}{v_{\min}}$.

In the LPV framework, the state system matrix (*A*, $B_{d}$) is usually assumed to be in a polyhedral form [33], i.e., represented by a scheduling parameter α associated with a time delay τ,

(18) $$\begin{array}{c} \left[\begin{array}{cc} A(\tau ) & B_{d}\left(\tau \right) \end{array}\right]\in \Omega =\sum_{i=1}^{2}\alpha^{i}\left(A_{i},B_{d,i}\right) \end{array}$$

where

(19) $$\begin{array}{c} \alpha \in \Delta \;≜\{\left(\alpha^{1},\alpha^{2}\right):\alpha^{i}\geq 0,\sum_{i=1}^{2}\alpha^{i}=1\},\\ \left(A_{1},B_{d,1}\right)=\left(A\left(\tau \right),B_{d}\left(\tau \right)\right){|}_{\tau =\tau_{1}}\\ \left(A_{2},B_{d,2}\right)=\left(A\left(\tau \right),B_{d}\left(\tau \right)\right){|}_{\tau =\tau_{2}} \end{array}$$

and gives the relationship between the scheduling parameter α and the online-measurable time delay τ.

(20) $$\begin{array}{c} \alpha^{1}=\left(\frac{1}{\tau_{2}}-\frac{1}{\tau }\right)/\left(\frac{1}{\tau_{2}}-\frac{1}{\tau_{1}}\right)\\ \alpha^{2}=\left(\frac{1}{\tau }-\frac{1}{\tau_{1}}\right)/\left(\frac{1}{\tau_{2}}-\frac{1}{\tau_{1}}\right) \end{array}$$

The basic idea in solving LPV systems is to transform the original problem into a min-max problem, i.e., to solve for the optimal solution of the cost function in the case where the scale parameters have the worst effect on the system. In this way, the optimal solution used satisfies the control constraints of the actual control process, regardless of the form of variation in the time-varying parameters. Thus, the optimization problem to be solved in the closed-loop MPC approach is

(21) $$\begin{array}{c} u\left(x_{k},\alpha_{k}\right)=\arg\min_{u_{k}}\max_{\alpha_{k}}J \end{array}$$

where *J* is the cost function of the optimization problem, and α(k) can be computed from information about the current available vehicle speed when combined with (10) and (20).

In MPC schemes [34], it is usually assumed that the cost function solves an optimization problem at time *k* with respect to a state prediction sequence of length $N_{p}$ of $\left\{x_{k+1},\cdots ,x_{k+N_{p}}\right\}$ and a future control action sequence of length $N_{c}$ of $\left\{u_{k},u_{k+1},\cdots ,u_{k+N_{c}-1}\right\}$ using the current time state $x_{k}$, the available time-varying parameter $\tau_{k}$, and the road preview information $W_{k}$. The cost function is therefore defined in this paper as

(22a) $$\begin{array}{cc} \min J(x_{k}) & \\ s.t.J\left(x_{k}\right) & =\sum_{i=1}^{N_{p}}\rho_{x}∥x\left(k+i\right){∥}_{\infty }+\sum_{i=0}^{N_{c}-1}\rho_{u}{∥u\left(k+i\right)∥}_{\infty } \end{array}$$

(22b) $$\begin{array}{cc} \; & x_{k+i+1}=A\left(\tau_{k+i}\right)x_{k+i}+B_{u}u_{k+i}\left(x_{k+i},\alpha_{k+i}\right)+B_{d}\left(\tau_{k+i}\right)w_{k+i}, \end{array}$$

(22c) $$\begin{array}{cc} \; & x_{\min}\leq x_{k}\leq x_{\max}, \end{array}$$

(22d) $$\begin{array}{cc} \; & u_{\min}\leq u_{k}\leq u_{\max} \end{array}$$

where the factor ρ is the objective function weight, and ${∥\cdot ∥}_{\infty }$ denotes the linear *∞*-norm.

Although the solution of (22) requires the search for an optimization solution for the next $N_{c}$ steps of the control action, only the first component $u^{*}\left(k\right)$ of the output optimization solution ultimately acts on the system, so the backward iterative solution method can effectively save the search computation of the optimization problem [35]. Since the time-varying parameter τ is only known at time i=0, only $\Theta ≜\{\tau \leq {\left[\tau_{2},-\tau_{1}\right]}^{T}\}$ is known for the remaining prediction times. Therefore, if the iterative process time of dynamic planning is k+i, the following problem must be considered:

(23a) $$\begin{array}{cc} \; & \min_{u}\max_{\alpha }J_{i}\left(x_{k+i}\right)=∥\rho_{x}x\left(k+i\right){∥}_{\infty }+{∥\rho_{u}u\left(k+i\right)∥}_{\infty }+J_{i+1}^{*}\left(x_{k+i+1}\right) \end{array}$$

(23b) $$s.t.x_{k+i+1}=A\left(\tau_{k+i}\right)x_{k+i}+B_{u}u_{k+i}\left(x_{k+i},\alpha_{k+i}\right)+B_{d}\left(\tau_{k+i}\right)w_{k+i},$$

(23c) $$\begin{array}{cc} \; & x_{\min}\leq x_{k}\leq x_{\max}, \end{array}$$

(23d) $$\begin{array}{cc} \; & u_{\min}\leq u_{k}\leq u_{\max} \end{array}$$

In a polyhedral representation of the LPV system (14), the optimal solution can be assumed to occur only at the vertex position of the scheduling parameter α, so we introduce an additional linear variable *t* to transfer the variables associated with the constraint so that a robust optimal solution can be computed by iteratively enumerating the scheduling parameter. This can be reformulated as the following optimization problem:

(24a) $$\min_{u_{k+i}}\varphi$$

(24b) $$s.t.\forall \alpha_{k+i}\in \Delta$$

(24c) $$\begin{array}{cc} \; & ∥\rho_{x}x_{k+i}{∥}_{\infty }+{∥\rho_{u}u_{k+i}∥}_{\infty }+J_{i+1}^{*}\left(x_{k+i+1}\right)\leq \varphi , \end{array}$$

(24d) $$\begin{array}{cc} \; & x_{\min}\leq x_{k}\leq x_{\max}, \end{array}$$

(24e) $$\begin{array}{cc} \; & u_{\min}\leq u_{k}\leq u_{\max} \end{array}$$

However, the key to the controller proposed in this paper is that the last step of the backward iterative process is different from the previous steps, because the vehicle speed information $v_{k}$ at the current moment is easily accessible, for example, by GPS or INS, and therefore, the knowledge of the scheduling parameter values $\alpha_{k}$ can be used (20) to improve the control performance. Since A(τ) and *x* form a bilinear constraint in (23), $B_{d}\left(\tau \right)$ and *w* also form a bilinear constraint, thus limiting the subsequent parameter solution options. Similar to the solution in [36,37], we replace the optimization problem by solving for the following uncontrolled successor variables, i.e.,

(25) $$\begin{array}{c} {\tilde{x}}_{k}=\left(\sum_{j=1}^{2}\alpha_{k}^{j}A_{j}\right)x_{k}\\ {\tilde{w}}_{k}=\left(\sum_{j=1}^{2}\alpha_{k}^{j}B_{d,j}\right)w_{k} \end{array}$$

We therefore replace the measurement status and road information in (14) by the uncontrolled successor variables in (25), i.e.,

(26) $$\begin{array}{c} x\left(k+1\right)={\tilde{x}}_{k}+B_{u}u\left(k\right)+{\tilde{w}}_{k} \end{array}$$

The solution to (24) can be obtained by transforming it into an mp-LP problem [38], which computes the optimal control law $u_{k}=f\left({\tilde{x}}_{k},\tau_{k},{\tilde{W}}_{k}\right)$,

(27) $$\begin{array}{c} f\left(\tilde{x},\tau ,\tilde{W}\right)=F^{i}\left(\tau \right)\left[\begin{array}{c} \tilde{x}\\ \tilde{W} \end{array}\right]+g^{i}\left(\tau \right)\\ \mathrm{if}\;H^{i}\left[\begin{array}{c} \tilde{x}\\ \tilde{W} \end{array}\right]\leq k^{i},i=1,2,\cdots ,r \end{array}$$

where ${\tilde{W}}_{k}=\left[\begin{array}{c} {\tilde{w}}_{k},{\tilde{w}}_{k+1},\cdots ,{\tilde{w}}_{N_{p}} \end{array}\right]$ denotes the road preview information for uncontrolled successor types. It is assumed that the preview information covers the entire prediction horizon, with *N* in Equation (16) being at least of size $N_{p}$. Here, *r* denotes the number of feasible solutions to the optimization problem (24).

### 3.2. State Estimation

The Kalman filter can accurately estimate the state of a vehicle suspension system with measurement noise and system process noise. This precise state estimation can serve as an input to the suspension MPC-LPV control algorithm, thereby computing the required control force. Therefore, the application of the Kalman filter in this control scheme is crucial, enabling the control algorithm to make timely adjustments based on accurate state estimates to counteract the effects of measurement noise and system process noise, thereby improving the control performance of the suspension system.

The basic idea of the standard Kalman filter is to solve for the optimal system state in the least-squares sense and to estimate the state of the suspension system by state prediction (28) and state correction (29) iterative models.

- Prediction process:

(28) $$\begin{array}{c} \left\{\begin{array}{c} {\hat{x}}_{k|k-1}=A\left(\tau \right)\;{\hat{x}}_{k-1}+B_{u}\;u_{k-1}\\ P_{k|k-1}=A\;P_{k-1|k-1}\;A^{T}+B_{d}\left(\tau \right)\;Q\;B_{d}{\left(\tau \right)}^{T}\\ A\left(\tau \right)=\sum_{i=1}^{2}\alpha^{i}A_{i}\\ B_{d}\left(\tau \right)=\sum_{i=1}^{2}\alpha^{i}B_{d,i} \end{array}\right. \end{array}$$

- Update process:

(29) $$\begin{array}{c} \left\{\begin{array}{c} K_{k}=\frac{P_{k|k-1}C^{T}}{CP_{k|k-1}C^{T}+R_{k}}\\ {\hat{x}}_{k}={\hat{x}}_{k|k-1}+K_{k}\left(y_{k}-C{\hat{x}}_{k|k-1}\right)\\ P_{k|k}=\left(I-K_{k}C\right)P_{k|k-1} \end{array}\right. \end{array}$$

where $\hat{x}$ is the approximate estimate of *x*, and *Q* and *R* are the process noise variance and the sensor measurement noise variance, respectively. In this paper, it is assumed that the process noise is caused only by the road excitation, so the variance *Q* has the properties of a standard normal distribution, i.e., $w∼N\left(0,Q\right)$.

For suspension systems, inaccurate measurements of the noise variance (*R*) often exhibit time-varying characteristics: i.e., the noise variance changes slowly with time. To improve the accuracy of *R* estimation, this paper uses an approach similar to [39]. In this case,

(30) $$\begin{array}{c} {\hat{R}}_{k}=\frac{1}{m}\sum_{i=1}^{m}v_{k-i}v_{k-i}^{T}+CP_{k|k}C^{T} \end{array}$$

where *m* denotes the size of the moving window, and the residual $v_{k}$ represents the difference between the true observations of the system and its estimated values, which can be defined as

(31) $$\begin{array}{c} v_{k}=y_{k}-C{\hat{x}}_{k} \end{array}$$

**Figure 4:**
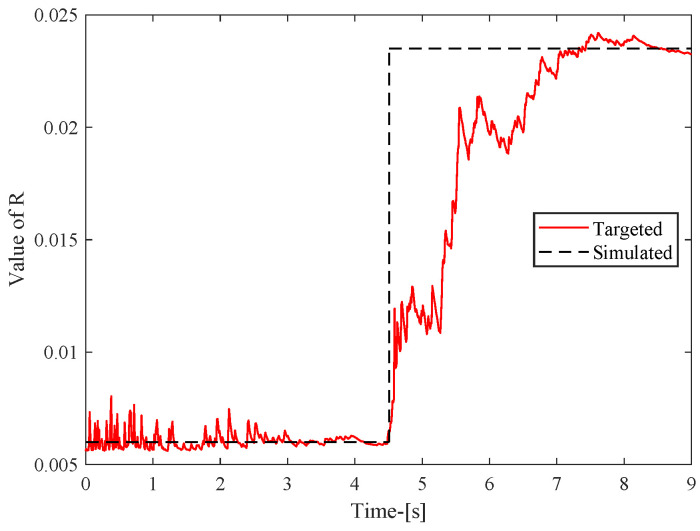
Estimated value of noise variance matrix R.

As can be seen from [40], ISO−B corresponds to an *R* value of 0.006, and ISO level C corresponds to an *R* value of 0.024. Using the above method, the convergence of *R* is shown in Figure 4.

## 4. Experiments and Results

We utilized MATLAB R2021b to validate the performance of the controller proposed in this paper. The MPLP problem was implemented using the YALMIP/Multi-Parametric Toolbox (MPT), with a sampling time of $T_{s}=0.01$ s. The parameters of the half-car model and the prediction and control steps of the MPC controller are listed in Table 1. The constraint of the front and rear actuators is set to u∈[−1500,1500]N.

**Figure 5:**
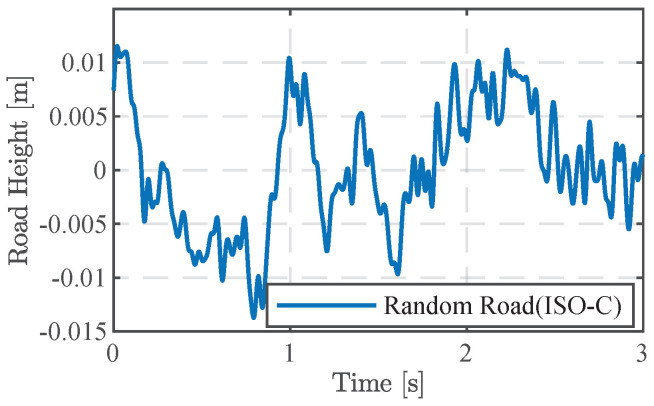
Random road.

In this section, we establish ISO level C random road surface input based on Equation (14) and $G_{q}\left(n_{0}\right)=256\times 10^{-6}\;m^{3}$ to observe the suspension response at different vehicle speeds, aiming to validate the improvement effect of the proposed MPC-LPV method under varying vehicle speeds. The road surface input is illustrated in Figure 5, and the improvement effects under different vehicle speed variations are compared. This paper introduces MPC-LPV, with its open-loop system employing only passive control (Passive) and a robust control algorithm (Robust).

**Figure 6:**
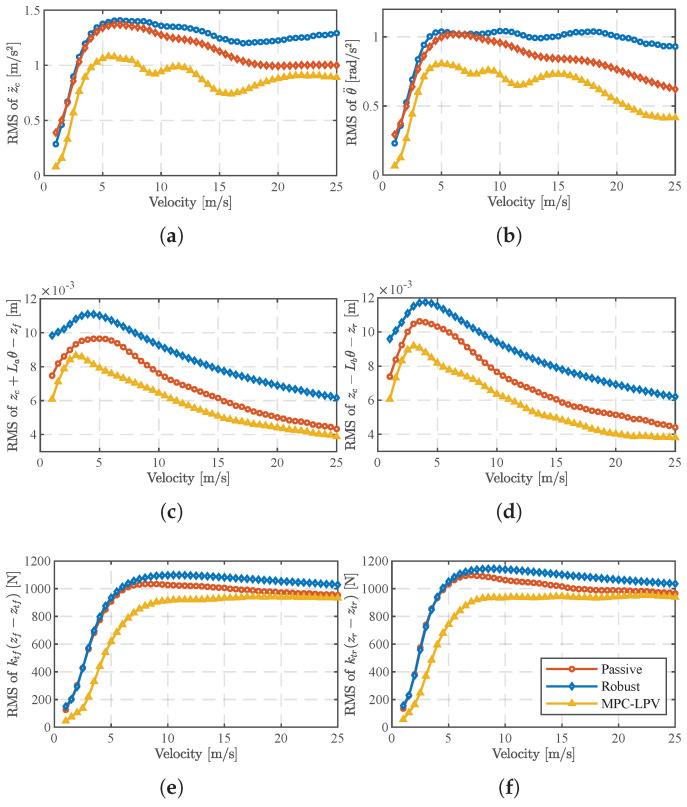
The RMS value of a suspension system on one bump road. (**a**) The RMS value of ${\ddot{z}}_{c}$. (**b**) The RMS value of $\ddot{\theta }$. (**c**) The RMS value of $z_{c}+L_{a}\theta -z_{f}$. (**d**) The RMS value of $z_{c}+L_{b}\theta -z_{r}$. (**e**) The RMS value of $k_{tf}\left(z_{f}-z_{tf}\right)$. (**f**) The RMS value of $k_{tr}\left(z_{r}-z_{tr}\right)$.

On random road surfaces, the proposed controller can account for the variation in vehicle speed and reduce the conservativeness of the controller optimization solution by using the vehicle speed information available at the current moment. Figure 6 shows the root mean square (RMS) of the suspension response for vehicle speeds from 1 m/s to 25 m/s, and the results show that the MPC-LPV controller outperforms the other control methods for different vehicle speed cases.

In Figure 6a,b, it is also found that, at low speeds (v< 5 m/s), the proposed MPC-LPV does not differ significantly from the open-loop system relying only on passive control in terms of RMS values for ${\ddot{z}}_{c},\ddot{\theta }$, with the main difference between the two concentrated at moderate speeds (5 m/s <v< 25 m/s). In Figure 6c,d, the suspension dynamic travel reaches its maximum at *v* = 5 m/s, and increasing the vehicle speed improves the suspension response in this category. In Figure 6e,f, the dynamic tire load remains essentially constant when the vehicle speed is greater than 12 m/s. This is because the experimental procedure assumes that the spring-loaded mass is constant and that the dynamic tire load is limited by the constraints of (7) to ensure driving safety, and even if the vehicle speed increases, the damping effect of MPC-LPV, which takes into account the effect of time-varying vehicle speed, does not violate the actual limiting constraint, and the advantage of the other controllers is gradually reduced.

**Figure 7:**
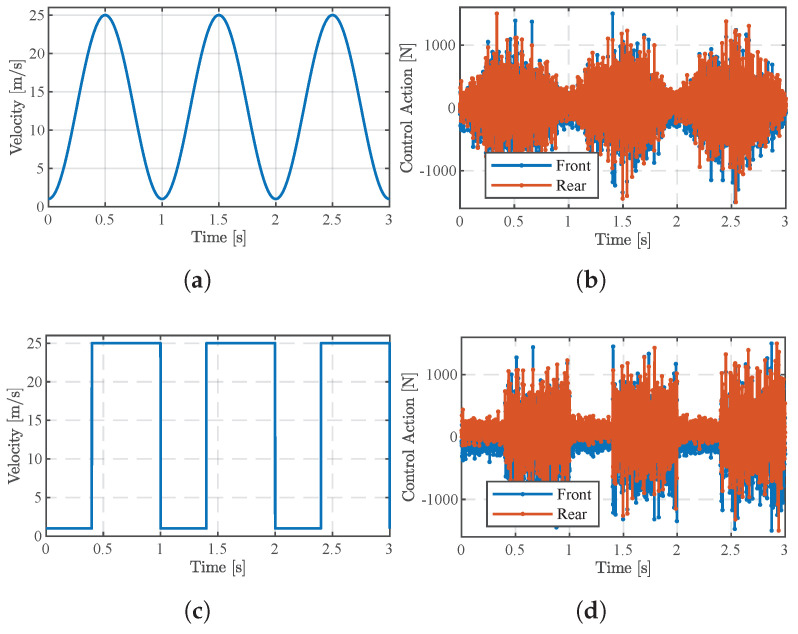
Active suspension control of force input at different speeds. (**a**) Velocity change for sinusoidal wave. (**b**) Control action corresponding to sinusoidal wave. (**c**) Velocity change for square wave. (**d**) Control action corresponding to square wave.

Furthermore, based on a random road surface, we tested the effect of the MPC-LPV controller with two cases with different velocity time steps, as shown in Figure 7. In case one, as shown in Figure 7a, the speed changes continuously and slowly with time steps in the range of 1∼25 m/s, i.e., sinusoidal input. In the second case, as shown in Figure 7c, the velocity varies intermittently, with a maximum value of 25 m/s and a minimum value of 1 m/s, i.e., square-wave input. The control force inputs corresponding to the two different cases of speed variation are shown in Figure 7c,d. It can be clearly seen that the trend of the control force input change is the same as the trend of the speed change, which shows that the MPC-LPV control pair proposed in this paper can provide a timely response to the speed change.

**Figure 8:**
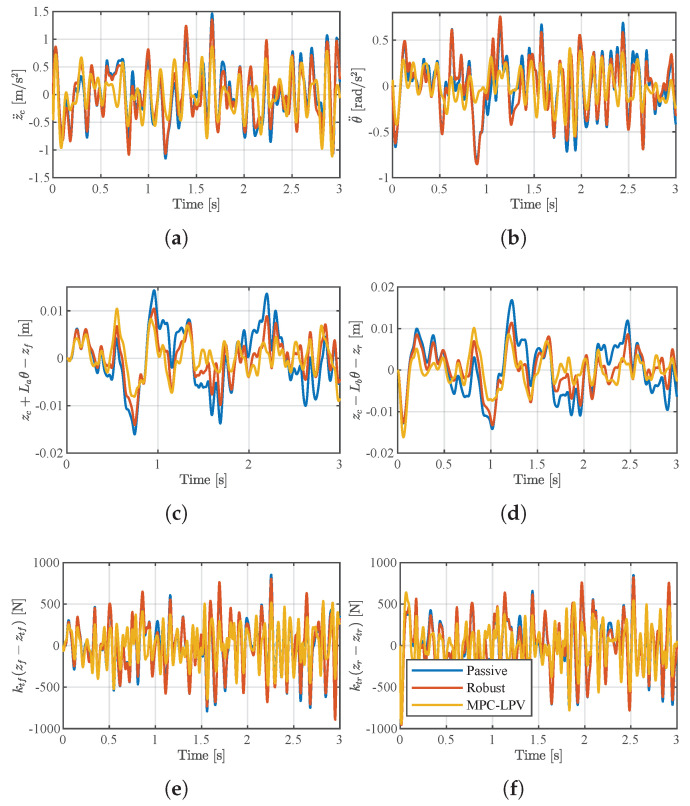
Responses under a random road profile. (**a**) The response value of $y_{1}$. (**b**) The response value of $y_{2}$. (**c**) The response value of $y_{3}$. (**d**) The response value of $y_{4}$. (**e**) The response value of $y_{5}$. (**f**) The response value of $y_{6}$.

Finally, the results of the dynamic response of the suspension in the speed variation case in Figure 7a are shown in Figure 8. In order to quantitatively demonstrate the superiority of the MPC-LPV controller proposed in this paper compared with other methods, we statistically calculated the corresponding RMS (Table 2) and peak-to-peak values (Table 3). It can be clearly seen that MPC-LPV has the smallest results compared to the passive suspension method, where the output response of the suspension system is reduced by 27% ($y_{1}$), 40% ($y_{2}$), 40% ($y_{3}$), 33% ($y_{4}$), 29% ($y_{5}$), and 21% ($y_{6}$) compared to passive suspension. The peak-to-peak values are 32% ($y_{1}$), 42% ($y_{2}$), 40% ($y_{3}$), 18% ($y_{4}$), 23% ($y_{5}$), and 8% ($y_{6}$) better than those for passive suspension.

## 5. Conclusions

In this study, we propose a novel approach to enhancing the vehicle suspension response through the application of model predictive control (MPC) based on a linear parameter-varying (LPV) framework. The key contributions of our work are as follows:

Model Reformulation: We redefined the half-car model by augmenting it with front- and rear-wheel time delays, utilizing the Padé approximation method to account for the time delay characteristics between these wheels. This reformulation enables a more accurate representation of the vehicle dynamics, particularly in relation to suspension behavior.

Noise Estimation Technique: We introduced a residual-vector-based variance matching technique to estimate time-varying noise in the suspension system. This technique enhances the accuracy of state estimation, providing more reliable feedback for control actions.

MPC-LPV Control Scheme: We designed MPC control schemes within the LPV model framework, incorporating road preview information and real-time vehicle speed data. By leveraging multi-parameter linear programming, we generated optimal control solutions offline, facilitating efficient online implementation.

Performance Evaluation: Simulation results across various road scenarios demonstrate the effectiveness of our proposed method in improving suspension performance compared to passive suspension systems. The MPC-LPV controller consistently outperforms conventional control methods, exhibiting superior response characteristics at different vehicle speeds and under different road conditions.

In conclusion, our study presents a comprehensive framework for enhancing the vehicle suspension response through MPC-LPV control. By leveraging advanced modeling techniques, noise estimation methods, and optimization algorithms, we achieved significant improvements in suspension performance. These findings underscore the potential of our approach for practical implementation, promising advancements in vehicle stability and maneuverability in real-world driving scenarios.

## Figures and Tables

**Table 1:** Parameters for half-car suspension model.

Symbol and Unit	Value	Symbol and Unit	Value
*M*-[kg]	500	*I*-[kg·m^2^]	910
$m_{f}$-[kg]	30	$m_{r}$-[kg]	40
$L_{a}$-[m]	1.25	$L_{b}$-[m]	1.45
$k_{f}$-[kN/m]	10	$k_{r}$-[kN/m]	10
$k_{tf}$-[kN/m]	100	$k_{tr}$-[kN/m]	100
$c_{f}$-[kN·s/m]	1	$c_{r}$-[kN·s/m]	1
$N_{p}$	10	$N_{c}$	3

**Table 2:** The comparison of RMS values in the suspension response.

	Passive	Robust	MPC-LPV
$y_{1}$	0.4633	0.4551	0.3382
$y_{2}$	0.3154	0.3095	0.1893
$y_{3}$	0.0065	0.0049	0.0039
$y_{4}$	0.0070	0.0054	0.0047
$y_{5}$	278.95	281.57	198.62
$y_{6}$	307.97	310.59	242.52

**Table 3:** The comparison of peak-to-peak values in the suspension response.

	Passive	Robust	MPC-LPV
$y_{1}$	2.6181	2.4375	1.7699
$y_{2}$	1.6047	1.4697	0.9229
$y_{3}$	0.0303	0.0246	0.0181
$y_{4}$	0.0310	0.0247	0.0253
$y_{5}$	1516.9	1497.8	1165.1
$y_{6}$	1689.1	1711.4	1550.9

## Data Availability

Dataset available on request from the authors.

## Appendix A. Specific Description of Each Type of Parameter Matrix

### Appendix A.1. Half-Car System Equation Matrix

(A1)
$$a_{1}=\frac{1}{M}+\frac{L_{a}^{2}}{I},a_{2}=\frac{1}{M}-\frac{L_{a}L_{b}}{I},a_{3}=\frac{1}{M}+\frac{L_{b}^{2}}{I}$$

(A2)
$$A_{c}=\left[\begin{array}{cc} 0_{4\times 4} & I_{4\times 4}\\ A_{1} & A_{2} \end{array}\right]$$

(A3)
$$A_{1}=\left[\begin{array}{cccc} -a_{1}k_{f} & -a_{2}k_{r} & a_{1}k_{f} & a_{2}k_{r}\\ -a_{2}k_{f} & -a_{3}k_{r} & a_{2}k_{f} & a_{3}k_{r}\\ \frac{k_{f}}{m_{f}} & 0 & -\frac{k_{f}+k_{tf}}{m_{f}} & 0\\ 0 & \frac{k_{r}}{m_{r}} & 0 & -\frac{k_{r}+k_{tr}}{m_{r}} \end{array}\right]$$

(A4)
$$A_{2}=\left[\begin{array}{cccc} -a_{1}c_{f} & -a_{2}c_{r} & a_{1}c_{f} & a_{2}c_{r}\\ -a_{2}c_{f} & -a_{3}c_{r} & a_{2}c_{f} & a_{3}c_{r}\\ \frac{c_{f}}{m_{f}} & 0 & -\frac{c_{f}}{m_{f}} & 0\\ 0 & \frac{c_{r}}{m_{r}} & 0 & -\frac{c_{r}}{m_{r}} \end{array}\right]$$

(A5)
$$B_{c,u}=\left[\begin{array}{cc} 0_{4\times 1} & 0_{4\times 1}\\ a_{1} & a_{2}\\ a_{2} & a_{3}\\ -\frac{1}{m_{f}} & 0\\ 0 & -\frac{1}{m_{r}} \end{array}\right],B_{c,d}=\left[\begin{array}{cc} 0_{6\times 1} & 0_{6\times 1}\\ \frac{k_{tf}}{m_{f}} & 0\\ 0 & \frac{k_{tr}}{m_{r}} \end{array}\right]$$

(A6)
$$C_{c}=\left[\begin{array}{cc} C_{1} & C_{2}\\ C_{3} & 0_{4\times 4} \end{array}\right]$$

(A7)
$$C_{1}=\left[\begin{array}{cccc} -\frac{k_{f}}{M} & -\frac{k_{r}}{M} & \frac{k_{f}}{M} & \frac{k_{r}}{M}\\ -\frac{L_{a}k_{f}}{I} & \frac{L_{b}k_{r}}{I} & \frac{L_{a}k_{f}}{I} & -\frac{L_{b}k_{r}}{I} \end{array}\right]$$

(A8)
$$C_{2}=\left[\begin{array}{cccc} -\frac{c_{f}}{M} & -\frac{c_{r}}{M} & \frac{c_{f}}{M} & \frac{c_{r}}{M}\\ -\frac{L_{a}c_{f}}{I} & \frac{L_{b}c_{r}}{I} & \frac{L_{a}c_{f}}{I} & -\frac{L_{b}c_{r}}{I} \end{array}\right]$$

(A9)
$$C_{3}=\left[\begin{array}{cccc} 1 & 0 & -1 & 0\\ 0 & 1 & 0 & -1\\ 0 & 0 & k_{tf} & 0\\ 0 & 0 & 0 & k_{tr} \end{array}\right]$$

(A10)
$$D_{c,u}=\left[\begin{array}{cc} \frac{1}{M} & \frac{1}{M}\\ \frac{L_{a}}{I} & -\frac{L_{b}}{I}\\ 0_{4\times 1} & 0_{4\times 1} \end{array}\right],D_{c,d}=\left[\begin{array}{cc} 0_{4\times 1} & 0_{4\times 1}\\ -k_{tf} & 0\\ 0 & -k_{tr} \end{array}\right]$$

All parameters are properly defined in Table 1.

### Appendix A.2. Front- and Rear-Wheel Road Input System Model

(A11) $$A_{\xi }=\left[\begin{array}{cccc} 0 & \frac{1}{\tau } & \cdots & 0\\ \vdots & \vdots & \ddots & \vdots \\ 0 & 0 & \cdots & \frac{1}{\tau }\\ -\frac{\beta_{0}}{\beta_{m}\tau } & -\frac{\beta_{1}}{b_{m}\tau } & \cdots & -\frac{\beta_{m-1}}{\beta_{m}\tau } \end{array}\right]$$

(A12) $$B_{\xi }=\left[\begin{array}{c} 0_{(m-1)\times 1}\\ \frac{1}{\beta_{m}\tau } \end{array}\right]$$

(A13) $$C_{\xi }={\left[\begin{array}{c} \left[{\left(-1\right)}^{0}-{\left(-1\right)}^{m}\right]\beta_{0}\\ {}[{\left(-1\right)}^{1}-{\left(-1\right)}^{m}]\beta_{1}\\ \vdots \\ {}[{\left(-1\right)}^{m-1}-{\left(-1\right)}^{m}]\beta_{m-1} \end{array}\right]}^{T}$$

(A14) $$D_{\xi }={\left(-1\right)}^{m}$$

where $\beta_{i}$ is properly defined in (13), *m* is the order of the Padé approximation, and in this paper, *m* = 2.

### Appendix A.3. Augmented System Model

$$\begin{array}{c} A=\left[\begin{array}{ccc} A_{c} & B_{c,d}\left[\begin{array}{c} 0\\ C_{\xi } \end{array}\right] & B_{c,d}\left[\begin{array}{c} I\\ D_{\xi } \end{array}\right]\\ 0 & A_{\xi } & B_{\xi }\\ 0 & 0 & -2\pi n_{1}v \end{array}\right],B_{u}=\left[\begin{array}{c} B_{c,u}\\ 0\\ 0 \end{array}\right],\\ B_{d}=\left[\begin{array}{c} 0\\ 0\\ 2\pi n_{0}\sqrt{G_{q}\left(n_{0}\right)v} \end{array}\right],C=\left[\begin{array}{c} C_{c},D_{c,d}\left[\begin{array}{c} 0\\ C_{\xi } \end{array}\right],D_{c,d}\left[\begin{array}{c} I\\ D_{\xi } \end{array}\right] \end{array}\right],\\ D_{u}=D_{c,u},D_{d}=0 \end{array}$$
